# A fibrosis-informed refinement of the intermediate-risk stratum for statin allocation: a liver-derived hypothesis anchored to the 2026 PREVENT-based guideline framework

**DOI:** 10.3389/fmed.2026.1926866

**Published:** 2026-08-25

**Authors:** Soon Woo Nam

**Affiliations:** Hepatobiliary Unit, Division of Gastroenterology, Department of Internal Medicine, Incheon St. Mary's Hospital, College of Medicine, The Catholic University of Korea, Seoul, Republic of Korea

**Keywords:** cardiovascular risk stratification, FIB-4, liver fibrosis, metabolic dysfunction-associated steatotic liver disease, number-needed-to-treat, PREVENT equations, primary prevention, statins

## Abstract

Statin efficacy is settled; the decisive question is in whom absolute benefit exceeds absolute harm by a margin justifying lifelong therapy. Because proportional risk reduction is approximately constant, absolute benefit scales with baseline risk while the principal harm, incident type 2 diabetes, does not, so a threshold must exist below which treatment is unjustified. The PREVENT equations, published in 2023–2024 and adopted by the 2026 ACC/AHA Multisociety dyslipidemia guideline, lower risk estimates and compress the intermediate band to a 10-year risk of 5% to less than 10%, a large, ambiguous stratum populated by patients with metabolic dysfunction-associated steatotic liver disease (MASLD). MASLD affects two in five adults, causes more cardiovascular than hepatic death, and is untreated in about half of eligible patients owing to a now-discredited fear of hepatotoxicity. Existing reviews establish that statins are safe in MASLD and that its cardiovascular risk is excess and graded, but none translates this into a tool for decision-making at the point of care. I advance a testable hypothesis: the fibrosis-4 (FIB-4) index and liver stiffness measurement should function as formal risk-enhancing modifiers subdividing this stratum. Every term of the upgrade rule is specified quantitatively: a two-threshold FIB-4 structure (rule-out below 1.3, or below 2.0 above age 65; rule-in at 2.67 or above, with 1.3–2.67 resolved by liver stiffness or coronary artery calcium), liver stiffness thresholds of 8 and 12 kPa, an explicit cardiometabolic-criterion list, and a 30-year PREVENT ASCVD risk of 10% or more. The supporting evidence is observational throughout, and I state plainly what is unestablished, namely that these markers add prognostic information beyond PREVENT and coronary artery calcium; fibrosis is framed as a marker of cumulative cardiometabolic injury, not a causal mediator. I situate the framework alongside resmetirom, incretin therapy, and bempedoic acid, define the fibrosis boundary beyond which competing liver-related mortality invalidates it, and specify a competing-risks validation design aligned to the PREVENT total cardiovascular disease endpoint, externally validated in East Asian and lean-MASLD cohorts. The contribution is a fully specified, falsifiable decision architecture for the stratum where guidelines leave clinicians most uncertain.

## 1 Introduction

Cardiovascular disease remains the leading cause of death worldwide, and statins are the most rigorously tested lipid-lowering agents, with an estimated 200 million users globally. Their efficacy is not in dispute. Pooled data from the Cholesterol Treatment Trialists' (CTT) Collaboration show that each 1 mmol/L (38.7 mg/dL) reduction in low-density lipoprotein cholesterol (LDL-C) produces an approximately one-fifth proportional reduction in major vascular events, a benefit that accumulates over time and is broadly consistent across subgroups, including individuals at lower baseline risk (1–3). Precisely because efficacy is settled, a recurrent argument reframes the statin from a prescription medicine into something closer to a public-health commodity. The fixed-dose polypill, proposed two decades ago, was projected to reduce cardiovascular events by more than 80% if given to all individuals above a certain age (4); the same reasoning animates contemporary proposals to reclassify statins as over-the-counter drugs, and, at its limit, to treat the drug as a population-level supplement rather than an individually indicated therapy (5). The appeal is clear: a cheap, generic, well-tolerated drug deployed as a near-universal preventive agent.

That position is nonetheless mistaken, and the nature of its error is instructive. The proportional benefit of statins is nearly invariant across the risk spectrum, so their absolute benefit is the arithmetic product of that fixed proportional reduction and the patient's pre-treatment absolute risk. Benefit therefore scales directly with baseline risk, whereas the drug's harms and costs are approximately fixed in absolute terms. The molecule that is near-mandatory for a 65-year-old with established atherosclerosis or familial hypercholesterolemia confers trivial absolute benefit, alongside real harm and cost, in a genuinely low-risk 40-year-old. The clinical question is thus never simply “does this drug work?” but “in whom does absolute benefit exceed absolute harm by a margin that justifies lifelong therapy?” The case against universal statinization is not a case against statins; it is a case for placing risk stratification, rather than the drug, at the center of clinical decision-making.

This argument has acquired new urgency and new precision in 2026. Two major documents, namely the inaugural cardiovascular-kidney-metabolic (CKM) syndrome guideline and a comprehensively revised ACC/AHA Multisociety dyslipidemia guideline, have both moved the field toward more granular, metabolically informed risk estimation rather than broader, undifferentiated prescription, and in doing so they have lowered the numeric treatment thresholds and compressed the pivotal “intermediate” band. I develop the consequences of that shift from a vantage point central to hepatology yet neglected in cardiology-led discussions: the liver. MASLD, the current nomenclature for what was previously called non-alcoholic fatty liver disease, is defined by the very cardiometabolic factors that drive atherosclerosis and affects an estimated 38% of adults worldwide (6, 7). Patients with MASLD more often die of cardiovascular disease than of liver disease, occupy some of the highest cardiovascular-risk strata, and are nonetheless among the least likely to receive a statin, largely because of a persistent and now-discredited fear of hepatotoxicity (8, 9).

My thesis is deliberately narrow and constructive. The liver does not merely raise the stakes of correct statin allocation; it supplies a cheap, routine, and biologically grounded variable, namely fibrosis, that may help resolve the newly compressed intermediate stratum. I therefore propose, as a falsifiable hypothesis, that noninvasive fibrosis markers be treated as formal risk-enhancing modifiers within the 2026 risk architecture, and I build an explicit, fully specified, testable rule around them. This article is a Hypothesis and Theory contribution: it presents previously posed positions accurately, offers a new interpretation of recent data, and advances a hypothesis that is testable within current knowledge.

## 2 The maximalist position, its grain of truth, and the gap this article fills

The maximalist position deserves fair statement before scrutiny. In its strongest form it runs as follows: because most statins are now generic and inexpensive, because LDL-C lowering is causally and dose-dependently related to event reduction, and because the principal barrier to population benefit is poor uptake and adherence rather than any lack of efficacy, broadening access, even through a fixed-dose polypill, could avert large numbers of events at the population level (4, 10, 11). Proponents further argue that statins compare favorably with aspirin on serious harm, that the under-treatment of genuinely high-risk patients is widespread and lethal, and that therapeutic timidity is the dominant clinical error. These points are individually defensible, and this article does not dispute them. The error lies in the inference from “statins benefit populations” to “statins should be given to nearly all individuals,” which silently conflates relative and absolute benefit.

A large and growing literature has examined statins in MASLD, and it is important to state precisely what that literature has and has not established, because the distinction defines this article's contribution. Recent narrative and systematic reviews have thoroughly summarized the safety and efficacy of statins in MASLD and other chronic liver diseases (7, 12), the cardiovascular burden of MASLD and the strategies available to reduce it (13), and the gradation of cardiovascular risk according to metabolic-factor burden within MASLD (14). Society guidance has independently affirmed statin safety across MASLD and compensated cirrhosis (8, 15), and observational and *post-hoc* analyses have linked statin exposure to improved hepatic outcomes (9, 16, 17). Collectively, this body of work answers two questions, namely whether statins are safe and useful in MASLD and whether cardiovascular risk in MASLD is elevated and graded, and it answers both affirmatively. What it does not do is convert those affirmations into a rule for the decision clinicians actually make: given a specific patient who now falls in the compressed 2026 intermediate band, should a statin be started today, and on what measurable, liver-derived basis? Table 1 makes this differentiation explicit.

**Table 1:** Positioning of this article relative to recent work on statins and MASLD.

Prior contribution	What it establishes	What it does not provide (the gap)
Reviews of statin safety/efficacy in MASLD [Commins et al. (7); Sharpton & Loomba (12)]	Statins are safe in MASLD and compensated cirrhosis; signals of hepatic benefit	No allocation rule; no link to the 2026 PREVENT bands; fibrosis treated as prognosis, not as a decision variable
Cardiovascular risk reduction in MASLD/MASH [Bernhard et al. (13)]	Frames CVD as the dominant threat in MASLD and reviews risk-reduction tools	Does not operationalize which intermediate-risk patient to treat, or on what threshold
Metabolic-burden gradation of CV risk in MASLD [Elsabaawy (14)]	Shows CV risk rises with metabolic-factor burden, sex, and age	Stops at association; no rule-based reclassification across a treatment threshold; not tied to PREVENT
FIB-4 and coronary atherosclerosis [Huang et al. (36); Lee & Lee (8)]	FIB-4 tracks prevalent and progressing coronary artery calcification in MASLD, with usable rule-in and rule-out thresholds	Reports diagnostic association with an imaging marker, not incremental prognostic value over PREVENT or a treatment rule
2026 dyslipidemia & CKM guidelines; PREVENT	Adopt PREVENT, lower thresholds, embed metabolic/renal biology; compress the intermediate band	Do not incorporate liver-derived fibrosis markers as risk-enhancing modifiers within the intermediate band
This article	Fibrosis (FIB-4, liver stiffness) as a formal, fully specified risk-enhancing modifier subdividing the 2026 PREVENT 5– < 10% band, with an explicit upgrade rule	Provides the missing procedural step and states it as a falsifiable hypothesis with a competing-risks validation design

CKM, cardiovascular-kidney-metabolic; CV, cardiovascular; FIB-4, fibrosis-4 index; MASLD, metabolic dysfunction-associated steatotic liver disease; MASH, metabolic dysfunction-associated steatohepatitis; PREVENT, Predicting Risk of cardiovascular disease EVENTs.

## 3 Why absolute risk, not efficacy, governs the decision

The proportional risk reduction conferred by statins is approximately constant across the risk spectrum: each 1 mmol/L LDL-C reduction lowers major vascular events by roughly 20–22%, whether baseline risk is high or low (1–3). Absolute benefit, however, is the product of this relative reduction and the patient's pre-treatment absolute risk, and therefore scales directly with that risk. The practical consequence is captured by the number-needed-to-treat (NNT): a patient with a 20% 10-year risk of atherosclerotic cardiovascular disease (ASCVD) derives several-fold greater absolute protection, with a correspondingly smaller NNT, than an otherwise identical patient at 5%, even though both experience the same proportional reduction. In low-risk primary prevention, the multi-year NNT becomes large and the expected per-patient gain small.

Harms, by contrast, are roughly constant in absolute terms and therefore weigh most heavily exactly where benefit is smallest. The best-characterized harm is new-onset type 2 diabetes (T2D). A collaborative meta-analysis of randomized trials demonstrated an approximately 9% relative increase in incident diabetes with statin therapy (odds ratio 1.09, 95% CI 1.02–1.17; one extra case per 255 patients treated for 4 years) (18), and a comparison of intensive vs. moderate-dose regimens estimated a number needed to harm per year of 498 for new-onset diabetes against a number needed to treat per year of 155 for a major cardiovascular event (19). This diabetogenic effect is dose-dependent and is concentrated in patients already near the dysglycemic threshold or carrying metabolic-syndrome features (20). Crucially, it is not merely an artifact of confounding: it tracks the on-target mechanism of the drug, because genetic variation that mimics HMG-CoA reductase inhibition is itself associated with modestly higher body weight and higher diabetes risk, anchoring the diabetogenic signal in pharmacology rather than chance (21).

**Figure 1:**
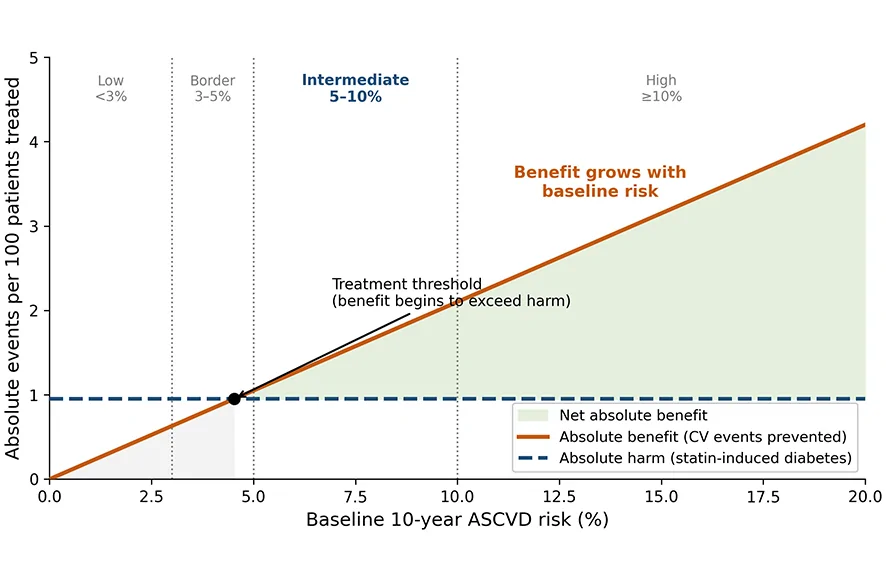
Why absolute risk, not efficacy, governs statin allocation. Because the proportional benefit of statins is approximately constant, absolute benefit (orange) rises with baseline risk, whereas the principal absolute harm, statin-induced diabetes (navy dashed), stays roughly fixed. Below the treatment threshold, at which the two lines cross, expected harm and cost exceed expected benefit, while above it net benefit widens. The schematic is illustrative and is not drawn to trial-derived scale. ASCVD, atherosclerotic cardiovascular disease.

Quantitatively, modeling across the 5%, 7.5%, and 10% treatment thresholds confirms that cardiovascular events prevented outnumber diabetes cases induced, with a likelihood of being helped vs. harmed (LHH) of approximately 2.3 to 2.9; yet the same modeling shows this margin narrowing, and even inverting, under higher estimates of statin-attributable diabetes risk (LHH 0.72–0.94), and reports systematically lower likelihood of benefit in women (LHH 1.74–2.40) and in adults aged 40–50 years (LHH 1.00–1.14) than in men (2.55–3.00) or adults aged 70–75 years (3.95–3.96) (22). Reassuringly, statin-associated diabetes does not appear to abolish net cardiovascular benefit in propensity-matched cohorts (23), and observational syntheses confirm that statin-associated harms are small in absolute terms relative to cardiovascular benefit among those most likely to gain (24). But the harm is not negligible, and it falls disproportionately on the metabolically vulnerable, who constitute the very MASLD population discussed below. This tension is not merely theoretical, and it has recently been quantified at the scale of a national population. Modeling the substitution of PREVENT for the pooled cohort equations across U.S. adults projects that approximately 53% of adults would be reclassified into a lower risk category, that 14.3 million (95% CI 12.6–15.9 million) fewer adults would be receiving or recommended for statin therapy, and that this contraction would produce approximately 107,000 additional myocardial infarctions or strokes over 10 years (25). That modeling did not quantify the offsetting reduction in statin-attributable diabetes, but the direction is not in doubt: narrowing eligibility trades ischaemic events for metabolic ones, and the entire clinical question is where along the risk spectrum the first quantity should be allowed to outweigh the second. The conclusion is structural rather than empirical: because benefit scales with baseline risk while harm and cost do not, a threshold of absolute risk must exist below which expected harm and cost exceed expected benefit. Identifying and refining that threshold, especially where the LHH is most finely balanced, is the entire clinical task, and the intermediate stratum is precisely where the balance is finest (Figure 1).

## 4 The 2026 stratification architecture: a compressed, metabolically informed middle

The 2018 AHA/ACC/Multisociety cholesterol guideline and the 2019 ACC/AHA primary-prevention guideline organized primary-prevention candidates by estimated 10-year ASCVD risk, using the pooled cohort equations (PCE), into four bands: low (< 5%), borderline (5% to < 7.5%), intermediate (≥7.5% to < 20%), and high (≥20%) (26, 27). High-risk patients warranted high-intensity therapy; intermediate-risk patients warranted moderate-to-high-intensity therapy through shared decision-making; borderline-risk patients warranted consideration when risk-enhancing factors were present; and low-risk patients were managed with lifestyle modification. Risk-enhancing factors such as premature family history, persistently elevated LDL-C or triglycerides, metabolic syndrome, chronic kidney disease, chronic inflammatory disease, and South Asian ancestry, together with coronary artery calcium (CAC) scoring, refined the decision in uncertain cases; a CAC score of zero predicted very low event rates and supported de-escalation (28, 29). The intermediate band remained explicitly the zone of greatest uncertainty and was large, encompassing approximately one quarter of U.S. adults (28).

Two developments between 2023 and 2026 reshaped this architecture, and both moved toward greater granularity rather than broader treatment. First, the PREVENT equations, developed and validated in 2023–2024, estimate 10- and 30-year risk of total cardiovascular disease, which now includes heart failure, from age 30, replace race with estimated glomerular filtration rate and metabolic variables, and operationalize the CKM construct (30, 31). It is worth stating precisely, because the point is easily blurred: the equations were not recalibrated in 2026; they were published in 2023–2024 and adopted, without re-derivation, by the 2026 guideline. Because PREVENT is better calibrated to contemporary populations, it yields systematically lower risk estimates than the PCE: applying it to existing thresholds would move approximately 53% of U.S. adults into a lower risk category and reduce the number eligible for or receiving statins by an estimated 14.3 million (25). Second, on 9 June 2026 the AHA, ACC, American Diabetes Association, and American Society of Nephrology jointly published the inaugural CKM syndrome guideline, staging patients 0–4 and embedding PREVENT-based risk into treatment decisions (32).

Most consequentially for the present argument, the 2026 ACC/AHA Multisociety dyslipidemia guideline now recommends PREVENT over the PCE for adults aged 30–79 and rebuilds the risk bands accordingly on the 10-year ASCVD estimate: low (< 3%), borderline (3% to < 5%), intermediate (5% to < 10%), and high (≥10%) (33). Within this scheme an intermediate-risk patient warrants at least a moderate-intensity statin, while CAC scoring resolves borderline-to-intermediate uncertainty: a CAC of 0 supports deferral, CAC >0 supports initiation, and CAC ≥100 or ≥75th percentile supports high-intensity therapy (33). The same guideline introduces a second, longitudinal pathway that is central to the rule proposed here: for adults aged 30–59, a 30-year PREVENT ASCVD risk of 10% or more is itself a basis for considering moderate-intensity statin therapy, independent of the 10-year estimate (33). This architecture matters for the present hypothesis in two ways. It lowers the numeric thresholds, so the “middle” I seek to refine is now the 5% to < 10% band rather than the legacy 7.5% to < 20% band; and, because PREVENT compresses predicted risk and narrows statin eligibility, it sharpens rather than softens the need to identify, within that compressed middle, the metabolically loaded patients in whom 10-year estimates understate lifetime hazard. Throughout the remainder of this article, “intermediate stratum” should be read in the 2026 PREVENT terms (5% to < 10%), with the legacy PCE band noted where continuity with prior literature requires it. The unifying principle across all three documents is identical: more granular, metabolically informed risk estimation rather than uniform prescription.

## 5 The liver as the highest-yield, least-served stratum

### 5.1 Phenotypic definitions and the cardiovascular primacy of MASLD

Because the terms in this field are often used interchangeably although they are not equivalent, I state at the outset which entity the proposed rule addresses. Hepatic steatosis is a finding, namely macrovesicular fat in at least 5% of hepatocytes or its imaging equivalent, and steatotic liver disease is the umbrella term for steatosis of any cause. MASLD is hepatic steatosis accompanied by at least one of five cardiometabolic criteria with other causes excluded, and MetALD is MASLD with increased alcohol intake as defined by the 2023 multisociety nomenclature (6). MASH is a histologic diagnosis requiring steatosis together with hepatocyte ballooning and lobular inflammation, and no noninvasive test establishes it. Fibrosis stage is likewise histologic, and noninvasive tests assign a risk category rather than a stage; that distinction is maintained throughout this article. Table 2 sets out each entity with its method of ascertainment, its prognostic implication, and its role, if any, in the rule proposed below.

**Table 2:** Phenotypic definitions in steatotic liver disease and their role in the proposed framework.

Term	Definition	How ascertained	Prognostic implication	Role in this framework
Hepatic steatosis	Macrovesicular fat in ≥5% of hepatocytes, or the imaging equivalent	Ultrasonography, computed tomography, MRI proton density fat fraction, controlled attenuation parameter, validated indices, or histology	Steatosis without metabolic dysfunction or fibrosis carries limited independent prognostic weight	A finding, not a diagnosis; never a trigger on its own
Steatotic liver disease (SLD)	Umbrella term for hepatic steatosis of any etiology	As above, with etiological work-up	Depends entirely on subtype	Umbrella term only; used when the subtype is not yet established
MASLD	Hepatic steatosis with at least one of five cardiometabolic criteria, other causes excluded, alcohol below the MetALD threshold	Imaging or histology for steatosis, with clinical and laboratory criteria	Associated with incident cardiovascular disease; cardiovascular rather than hepatic death predominates	The population to which the rule applies; two or more criteria are required for upgrade
MetALD	MASLD with increased alcohol intake as defined by the 2023 multisociety nomenclature	As MASLD, with quantified alcohol intake	Distinct trajectory; alcohol independently affects hepatic and cardiovascular outcomes	Not addressed by the rule; alcohol intake must be quantified and reported in any validation
MASH	MASLD with steatohepatitis, namely steatosis with hepatocyte ballooning and lobular inflammation	Histology only; no noninvasive test establishes it	Higher likelihood of fibrosis progression; the enrolment phenotype of the resmetirom and semaglutide trials	Deliberately excluded as a decision variable: not obtainable at the point of allocation, and fibrosis rather than steatohepatitis carries the prognostic weight
Fibrosis stage (F0–F4)	Histologic stage of hepatic fibrosis	Biopsy; noninvasive tests estimate a risk category, not a stage	The dominant determinant of liver-related and all-cause mortality in MASLD	The rule's exposure variable, used as a noninvasive risk category and not as a histologic stage
Compensated advanced chronic liver disease and cirrhosis	Advanced fibrosis or cirrhosis without decompensation, and decompensated cirrhosis	LSM ≥15 kPa, or ≥10 kPa with platelets < 150 × 10?/L, or cirrhosis on imaging or biopsy; decompensation defined clinically	Liver-related mortality becomes a major competing risk	Outside the framework; see section 7.5

MASH, metabolic dysfunction-associated steatohepatitis; MASLD, metabolic dysfunction-associated steatotic liver disease; MetALD, metabolic dysfunction-associated steatotic liver disease with increased alcohol intake; MRI, magnetic resonance imaging; SLD, steatotic liver disease. Noninvasive tests assign a risk category rather than a histologic stage.

Two consequences follow for the framework. Its target population is MASLD, and its exposure variable is a noninvasive fibrosis risk category rather than a histologic stage. MASH is deliberately excluded as a decision variable, for two reasons worth stating rather than assuming: it cannot be established without biopsy and is therefore unavailable at the point at which a statin decision is made in primary prevention, and within MASLD it is fibrosis rather than steatohepatitis that carries the prognostic weight, so a rule built on fibrosis category gains nothing from a phenotype that cannot be measured.

MASLD is now the most prevalent chronic liver disease, with an estimated global prevalence of approximately 38% in adults (7). Its cardiovascular relevance is twofold. First, MASLD is independently associated with incident cardiovascular disease, with a meta-analytic odds ratio of 1.64 (95% CI 1.26–2.13), rising to 2.58 (1.78–3.75) in more severe disease, and cardiovascular events, rather than liver disease, are the leading cause of death in this population (7, 34). That meta-analysis pooled 16 observational studies of 34,043 adults in which fatty liver was diagnosed on imaging or histology, and its authors state that the observational design of the included studies does not permit causal inference; the same caution applies to every association reported in this article (34). Second, cardiovascular risk within MASLD is itself stratified: greater metabolic burden, male sex, and older age are independent predictors of intermediate-to-high ASCVD risk (14). MASLD is therefore not a single stratum but a gradient, mapping naturally onto the borderline–intermediate–high architecture described above. Even so, many statin-eligible adults at borderline or intermediate risk remain untreated, a shortfall that MASLD only magnifies (35).

### 5.2 Dismantling the hepatotoxicity myth

Historical reluctance to prescribe statins in liver disease, rooted in fear of transaminase elevation, is unsupported by current evidence. The 2024 EASL–EASD–EASO MASLD guideline and the AASLD practice guidance both state that statins are safe in adults with MASLD and with compensated cirrhosis and should be prescribed according to cardiovascular risk guidelines; asymptomatic aminotransferase elevation is not a contraindication, and routine enzyme monitoring solely for hepatotoxicity surveillance is no longer recommended (8, 15). Caution remains appropriate only in decompensated cirrhosis, where pharmacokinetics and the competing risk of muscle toxicity are less well characterized. Separately, and on a weaker evidential footing, a body of observational and *post-hoc* data associates statin use with lower rates of fibrosis progression, MASH, hepatic decompensation, and hepatocellular carcinoma, and with lower liver-related mortality, with the strongest signals in patients with elevated alanine aminotransferase (9, 12, 16). These hepatic signals are supportive context, not part of the case advanced here; the argument of this article rests entirely on the cardiovascular indication, which is established by randomized evidence.

### 5.3 Mechanistic humility: fibrosis as marker, not mediator

Two claims must be kept separate, and the literature frequently conflates them. The first is that hepatic fibrosis identifies patients at higher cardiovascular risk; this is well supported observationally. The second is that fibrosis causes that risk, or that reversing it would lower cardiovascular events; this is not supported. The more parsimonious interpretation, and the one adopted throughout this article, is that fibrosis is an integrated biomarker of cumulative cardiometabolic injury: it records the duration and intensity of insulin resistance, visceral adiposity, dyslipidemia, and inflammation that a single cross-sectional risk equation captures only imperfectly. Genetic evidence supports this reading in the reverse direction as well: in Mendelian randomization analyses, genetically proxied lipid-lowering drug-target effects were not clearly associated with reductions in liver fat, whereas several non-lipid metabolic traits were (36). Observational associations between statin exposure and hepatic outcomes may therefore reflect healthy-user bias, confounding by indication, or mechanisms downstream of rather than identical to on-target LDL lowering.

The honest conclusion is threefold. Current data do not establish an independent hepatic indication for statin therapy. They do decisively eliminate any rationale for withholding a cardiovascularly indicated statin in MASLD. And they justify using fibrosis as a risk marker, not as a therapeutic target, within a cardiovascular allocation rule. Language in the remainder of this article is chosen accordingly: fibrosis markers index risk, flag patients, and reclassify strata; they are not described as driving, causing, or mediating atherosclerotic events.

### 5.4 Fibrosis markers as candidate risk-enhancing modifiers: thresholds and their evidence

The hepatologist's distinctive contribution to stratification is the routine, inexpensive quantification of fibrosis, which is the variable that most strongly governs hepatic prognosis in MASLD and is associated with cardiovascular prognosis as well. Noninvasive tools, namely the fibrosis-4 (FIB-4) index, the NAFLD fibrosis score, and vibration-controlled transient elastography (liver stiffness measurement, LSM), already triage patients in hepatology clinics and are directly portable to cardiovascular decision-making; FIB-4 in particular is computed from age, aminotransferases, and platelet count, so it is available at essentially no marginal cost in any patient who has had routine blood work.

The question is which numeric thresholds are defensible, and on what basis. FIB-4 ≥1.3 is a rule-out threshold for advanced hepatic fibrosis, not a validated marker of elevated cardiovascular risk, and it should not be used alone to move a patient across a treatment threshold. I therefore replace the single cut-point used in earlier formulations of this proposal with a two-threshold structure that mirrors how the index actually performs. In a MASLD cohort undergoing coronary computed tomography, a FIB-4 of 1.26 gave the best negative predictive value for severe coronary artery calcification (95.1%), whereas a FIB-4 ≥2.67 gave the highest positive predictive value for the presence of any coronary artery calcification (89.7%); the authors accordingly proposed FIB-4 as a referral index for coronary imaging (37). In an independent longitudinal cohort of 60,445 asymptomatic Korean adults undergoing serial coronary computed tomography between 2012 and 2023, a time-updated FIB-4 was associated with progression of coronary artery calcification in MASLD, chiefly in men and in those older than 40 years; the authors themselves characterize the association as modest and describe FIB-4 as an accessible marker of subclinical cardiovascular risk rather than a validated means of improving risk stratification (38). At the level of pooled epidemiology, a meta-analysis of 19 cohorts comprising 1,481,875 adults found FIB-4 associated with incident cardiovascular events (odds ratio 1.77, 95% CI 1.58–1.99; per one-unit increment, odds ratio 1.21, 95% CI 1.12–1.31), with the certainty of evidence graded low (39). That meta-analysis also reports a finding that bears directly on the interpretation advanced here: the association was essentially the same in adults with and without NAFLD (odds ratio 1.87 vs. 1.74; P for difference 0.56), which argues that FIB-4 indexes systemic cardiometabolic risk rather than anything liver-specific. In the UK Biobank, noninvasive fibrosis scores were associated with incident heart failure among 413,860 adults over a median of 10.7 years (FIB-4 hazard ratio 1.69, 95% CI 1.55–1.84), a component of the PREVENT composite endpoint that coronary calcium does not capture (40).

The provenance of this evidence should be stated explicitly, because the thresholds proposed below inherit it. For each supporting, the design, the method by which steatosis and fibrosis were ascertained, the cardiovascular outcome and how it was determined, whether events were prevalent or incident, and the adjustment made. Three features deserve emphasis. None of the studies from which the thresholds are drawn staged fibrosis histologically: in the coronary computed tomography cohort, steatotic liver disease was identified by ultrasonography or a hepatic steatosis index above 36 and fibrosis was represented by FIB-4 alone, with neither elastography nor biopsy (37); in the longitudinal Korean cohort, steatosis was ascertained by ultrasonography and fibrosis by a time-updated FIB-4 (38); and in the UK Biobank analysis and the pooled meta-analysis, fibrosis was represented by serum scores only (39, 40). The two studies that supply the FIB-4 cut-points did not measure clinical events but coronary artery calcification, which is an imaging surrogate, and in the first of them the design is cross-sectional and the calcification prevalent, so the positive predictive value of 89.7% is a statement about prevalent calcification in a health-check population rather than about incident events (37). Adjudicated or registry-linked clinical endpoints appear only in the UK Biobank analysis, in which 12,527 incident hospitalizations for or deaths from heart failure were ascertained through linked records (40), and in the pooled cohorts, in which cardiovascular events were defined as cardiovascular mortality, myocardial infarction, and coronary heart disease, an outcome that excludes heart failure and therefore does not correspond to the composite endpoint adopted in section 8 (39). No study in this literature has used an independent, blinded endpoint adjudication committee.

Three operational thresholds follow, and each is stated as a default to be tested rather than a validated cut-point. First, FIB-4 below 1.3 defines the rule-out zone, in which the liver adds nothing to the cardiovascular calculus. Second, FIB-4 of 2.67 or above defines the rule-in zone, at which the index carries a high positive predictive value for prevalent coronary calcification and, in the framework proposed below, is sufficient on its own as the marker of advanced biology. Third, the intermediate FIB-4 zone of 1.3 to 2.67, which in practice is where most MASLD patients fall, is explicitly indeterminate and must be resolved by a second test, either liver stiffness measurement or coronary artery calcium, rather than acted upon directly. For liver stiffness I adopt the thresholds of the 2024 EASL–EASD–EASO guideline: below 8 kPa indicates low risk of advanced fibrosis, 8 to 12 kPa is indeterminate, and above 12 kPa indicates high risk (8). Only LSM above 12 kPa qualifies as a marker of advanced biology in the rule below. Each of these values is either a hepatic diagnostic threshold or a threshold derived against an imaging surrogate, and none has been derived against an adjudicated cardiovascular endpoint; that is the sense in which they are defaults to be tested rather than validated cut-points.

Age requires a separate provision, because FIB-4 loses specificity in older adults in whom age itself inflates the numerator. In a multicentre study of biopsy-proven NAFLD, the specificity of FIB-4 for advanced fibrosis fell to 35% beyond age 65, and a derived and validated cut-off of 2.0 restored specificity to 70% without loss of sensitivity (77%) in that group; below age 35 the index performed no better than chance (area under the curve 0.60) (41). Accordingly, in adults aged 65 years and older the rule-out threshold becomes FIB-4 < 2.0, a FIB-4 between 2.0 and 2.67 is indeterminate and requires liver stiffness or coronary calcium, and FIB-4 ≥2.67 retains its rule-in status. In adults younger than 35 years FIB-4 also performs poorly and should not be used as the sole modifier. These provisions are summarized, with every other term of the rule, in Table 3.

**Table 3:** Operational definitions for every term of the proposed upgrade rule.

Term	Operational definition	Basis and status
Intermediate stratum	PREVENT 10-year ASCVD risk 5% to < 10%, adults aged 30–79	2026 ACC/AHA Multisociety dyslipidemia guideline; adopted definition
MASLD	Hepatic steatosis on imaging or biopsy plus ≥1 cardiometabolic criterion, other causes excluded	2023 multisociety nomenclature; adopted definition
≥2 cardiometabolic criteria	Two or more of: (i) BMI ≥25 kg/m^2^ (≥23 in Asian populations) or waist circumference >94 cm (M)/>80 cm (F); (ii) fasting glucose ≥5.6 mmol/L, 2-h post-load ≥7.8 mmol/L, HbA1c ≥5.7%, type 2 diabetes, or treatment for it; (iii) blood pressure ≥130/85 mmHg or antihypertensive therapy; (iv) plasma triglycerides ≥1.70 mmol/L or lipid-lowering therapy; (v) HDL-C ≤ 1.0 mmol/L (M)/ ≤ 1.3 mmol/L (F) or lipid-lowering therapy	2023 multisociety nomenclature; adopted definition
FIB-4 rule-out	< 1.3 (age < 65); < 2.0 (age ≥65). Not applicable as sole modifier below age 35	Age-specific performance of FIB-4 in biopsy-proven NAFLD; adopted from hepatology practice
FIB-4 indeterminate	1.3–2.67 (age < 65); 2.0–2.67 (age ≥65). Requires LSM or CAC to resolve; does not satisfy the rule alone	Proposed default; the zone in which most MASLD patients fall
FIB-4 rule-in	≥2.67	Highest positive predictive value (89.7%) for prevalent coronary artery calcification in MASLD; proposed default requiring prospective testing
Elevated liver stiffness	VCTE >12 kPa (8–12 kPa indeterminate; < 8 kPa rule-out)	EASL–EASD–EASO 2024 risk categories for advanced fibrosis; adopted definition
High 30-year PREVENT risk	30-year PREVENT ASCVD risk ≥10% in an adult aged 30–59	2026 guideline threshold for considering moderate-intensity statin therapy; adopted definition
Marker of advanced biology	Any one of: FIB-4 rule-in; LSM >12 kPa; CAC >0; high 30-year PREVENT risk	Composite proposed here; the object of the validation programme in section 8
Upgrade	Manage as high-risk: initiate statin; high-intensity if CAC ≥100 or ≥75th percentile	Proposed hypothesis, not a validated decision rule

BMI, body-mass index; CAC, coronary artery calcium; CVD, cardiovascular disease; F, female; FIB-4, fibrosis-4 index; HbA1c, glycated hemoglobin; HDL-C, high-density lipoprotein cholesterol; LSM, liver stiffness measurement; M, male; MASLD, metabolic dysfunction-associated steatotic liver disease; PREVENT, Predicting Risk of cardiovascular disease EVENTs; VCTE, vibration-controlled transient elastography. Thresholds labeled “proposed default” are conceptual starting values for prospective testing, not validated cut-points.

### 5.5 What fibrosis markers would have to add, and to what: the incremental-value question

A framework of this kind is only worth adopting if fibrosis markers carry information that existing instruments do not. Two comparators matter, and the honest answer differs for each.

The first comparator is the PREVENT equations themselves. PREVENT already incorporates age, systolic blood pressure, total and HDL cholesterol, diabetes and glycated hemoglobin, smoking, body-mass index, and estimated glomerular filtration rate with urinary albumin-to-creatinine ratio (30). Every one of these is correlated with MASLD and with hepatic fibrosis, so a substantial share of the crude association between FIB-4 and cardiovascular events must be shared rather than incremental variance. The overlap is structural rather than incidental: age, the strongest single determinant of the PREVENT estimate, sits in the numerator of FIB-4 itself, and the remaining PREVENT inputs are the same variables that define MASLD. The association of FIB-4 with cardiovascular events was, moreover, essentially identical in adults with and without fatty liver disease, which argues that the index reflects systemic cardiometabolic burden rather than anything liver-specific and further raises the bar for any claim of independence (39). The published evidence is consistent with partial, not complete, overlap: FIB-4 remains associated with cardiovascular events after multivariable adjustment in large cohorts (39), and at least part of its association with mortality in high-cardiovascular-risk populations appears to be mediated through declining glomerular filtration, which PREVENT already measures. No study to date has reported the quantity that actually matters, namely the change in discrimination, calibration, and net reclassification when FIB-4 or LSM is added to PREVENT within the 5% to < 10% band. I state plainly that this is unproven, and I specify it in section 8 as the primary test of the hypothesis. What the fibrosis markers plausibly contribute, and what a single cross-sectional equation structurally cannot, is a record of cumulative exposure: two patients with identical current blood pressure, lipids, and glycaemia may have accumulated very different metabolic burden over two decades, and fibrosis is one of the few routinely measured variables that integrates that history.

The second comparator is coronary artery calcium, which the 2026 guideline already designates as the instrument for resolving intermediate-risk uncertainty (33). I do not claim that a fibrosis marker outperforms CAC as a measure of atherosclerotic burden; CAC images the disease itself, whereas FIB-4 indexes the metabolic milieu that produces it. The claim is narrower and, I would argue, more useful. FIB-4 costs nothing and is computable retrospectively in essentially every patient who has had a complete blood count and liver panel, whereas CAC requires a computed tomography scan that is not universally available, not universally reimbursed, and, in many health systems outside the United States, not routinely offered in primary prevention. Positioning FIB-4 upstream of CAC as a zero-cost referral filter, rather than as a competitor to it, is precisely the use proposed by the authors who characterized FIB-4 as a referral index for coronary calcification (37). In that role the relevant question is not whether FIB-4 beats CAC but whether FIB-4 identifies, at no cost, the intermediate-band patients in whom CAC is most likely to be informative, and whether it does so better than the metabolic variables already in PREVENT.

Discordance must nonetheless be handled explicitly, because it is common and the rule is otherwise ambiguous. Where a patient has an elevated FIB-4 but a CAC of zero, the CAC result should govern the atherosclerotic decision: the negative predictive value of a zero score is the best-established finding in this area (28, 29), and the 2026 guideline treats it as grounds for deferral (33). The elevated FIB-4 is not thereby discarded; it should trigger the hepatology fibrosis pathway on its own merits, shorten the interval to cardiovascular reassessment to 2–3 years rather than five, and raise attention to heart failure, which is included in the PREVENT composite endpoint, is associated with fibrosis scores in prospective data (40), and is not measured by coronary calcium. Where FIB-4 is low but CAC is above zero, the CAC again governs and statin therapy is indicated. The fibrosis marker therefore changes management independently of CAC in two defined situations: when CAC is unavailable or declined, and when the question is the timing and intensity of reassessment rather than the immediate binary decision. The worked example in section 7.3 is constructed around the first of these.

The magnitude of any incremental gain should also be anticipated realistically rather than assumed. The developers of PREVENT reported that adding urinary albumin-to-creatinine ratio, glycated hemoglobin, and a social deprivation index together improved discrimination for total cardiovascular disease by a change in the C-statistic of only 0.004 to 0.005 (30). A single serum index computed from variables already correlated with the equation's inputs should not be expected to exceed that. If this framework has value, it will appear as improved calibration and positive net benefit within the 5% to < 10% band rather than as a visible gain in global discrimination, and the validation specified in section 8 is designed accordingly.

### 5.6 Calibration in East Asian and lean-MASLD populations

Two phenotypic caveats deserve emphasis, both directly relevant to non-Western practice. First, lean MASLD, which is steatotic liver disease at a normal body-mass index and is disproportionately common in Asian populations, can carry substantial cardiometabolic risk despite an unremarkable habitus, and may be overlooked by clinicians anchored on obesity. Second, the PREVENT equations were derived predominantly in U.S. cohorts and deliberately removed race as an input; their calibration in East Asian and other non-Western populations, in whom MASLD prevalence and body-composition thresholds differ, remains to be confirmed. Applying Western numeric thresholds uncritically to Asian MASLD patients risks systematic under-treatment, and argues for using liver-derived modifiers, which are biologically rather than demographically defined, as a corrective. That the largest longitudinal dataset linking FIB-4 to coronary calcification progression comes from a Korean cohort is encouraging in this respect (38), though it also underlines that the fibrosis thresholds themselves may require population-specific calibration rather than direct transfer.

Despite carrying some of the highest cardiovascular risk, roughly half of statin-eligible patients with MASLD, and in some cohorts up to two-thirds, remain untreated (8, 9). In this population the dominant error is clearly under-treatment, the precise stratum in which maximalist advocates are correct that practice is too timid. The remedy, however, is not undifferentiated prescription; it is accurate stratification that recognizes MASLD, especially when accompanied by heavy metabolic burden and measurable fibrosis, as a risk-enhancing and often risk-defining condition. The liver thus simultaneously identifies where boldness is warranted and supplies the variables of metabolic load, fibrosis stage, age, and sex that are needed to calibrate it.

## 6 Statins in the era of disease-modifying MASH therapy

A 2026 account of statin allocation can no longer treat the statin as the only relevant drug in the MASLD patient, because the therapeutic landscape has changed abruptly. Resmetirom, a liver-directed thyroid hormone receptor-β agonist, became in 2024 the first agent approved for non-cirrhotic MASH with moderate-to-advanced fibrosis, achieving MASH resolution and fibrosis improvement vs. placebo in a phase 3 trial (42). In 2025, the phase 3 ESSENCE trial showed that the glucagon-like peptide-1 (GLP-1) receptor agonist semaglutide resolved steatohepatitis and improved fibrosis in patients with MASH (43). Both trials enrolled patients with biopsy-confirmed, non-cirrhotic steatohepatitis and significant fibrosis, a narrower and histologically defined phenotype than the population addressed by the rule proposed here, and their results should not be read as evidence about MASLD at large. These agents modify the liver disease directly; crucially, however, neither is a cardiovascular-risk-reduction therapy in the sense that statins are, and neither displaces the statin as the backbone of atherosclerotic risk reduction in MASLD. If anything, the arrival of effective hepatic therapy strengthens the argument for concurrent, guideline-indicated statin treatment, because patients are now identified, staged, and engaged in hepatology care at exactly the moment their cardiovascular risk should be addressed. It also introduces a caveat for the framework proposed here: if fibrosis becomes modifiable, a fibrosis marker measured after treatment no longer indexes cumulative lifetime metabolic exposure in the way an untreated measurement does, and the rule below should be applied to baseline rather than post-treatment values until this is studied.

The incretin therapies carry an additional, underappreciated implication for the central tension of this article. Because the principal harm of statins, which is incident diabetes, is concentrated in the metabolically vulnerable MASLD patient, the increasing co-prescription of GLP-1 receptor agonists, which improve glycemia and weight, may partially offset the diabetogenic cost of statin therapy in precisely the subgroup where it weighs most. Conversely, for the intermediate-risk MASLD patient in whom diabetogenicity is the dominant hesitation and a statin is poorly tolerated, bempedoic acid offers an instructive alternative: in the CLEAR Outcomes trial it reduced major adverse cardiovascular events in statin-intolerant patients (11.7% vs. 13.3%; hazard ratio 0.87, 95% CI 0.79–0.96), and the excess adverse events attributed to it were gout and cholelithiasis rather than dysglycaemia (44). Its metabolic neutrality has since been examined directly rather than inferred: a prespecified analysis of CLEAR Outcomes reported no increase in new-onset diabetes and no worsening of glycated hemoglobin among participants without diabetes at baseline (45), and a patient-level pooled analysis of four phase 3 trials found annual rates of new-onset diabetes of 0.3% with bempedoic acid vs. 0.8% with placebo among normoglycaemic participants and 4.7% vs. 5.9% among those with prediabetes, alongside small reductions rather than increases in glycated hemoglobin (46). A drug that lowers LDL-C and cardiovascular risk without the diabetogenic penalty is conceptually well suited to the metabolically loaded middle stratum, and offers a concrete escape valve within the upgrade rule proposed below.

## 7 The hypothesis: a fibrosis-informed refinement of the strata

If the statin is a risk-stratified drug, improving care requires improving the strata. I advance three linked recommendations, expressed in the 2026 PREVENT-based bands, of which the second is the core hypothesis of this article. Section 7.5 then states the boundary beyond which the framework does not apply.

### 7.1 Endorse subdivision of the high-risk stratum

I endorse finer subdivision within the high-risk tier. The distinction between very-high-risk and high-risk patients, in which the former encompasses recurrent events, multivessel atherosclerosis, familial hypercholesterolemia, diabetes with target-organ damage, or very-high-risk chronic kidney disease, should drive correspondingly intensive LDL-C targets and earlier combination therapy with ezetimibe or PCSK9 inhibition. The 2026 CKM staging system, in which stages 3 and 4 capture subclinical and clinical cardiovascular disease in the setting of metabolic and renal dysfunction, provides a ready scaffold for this (32). From a hepatological standpoint, MASLD with advanced fibrosis combined with diabetes belongs firmly in this very-high-risk group, subject to the boundary conditions set out in section 7.5.

### 7.2 Subdivide the intermediate stratum by an explicit, liver-derived rule

The intermediate band (5% to < 10% 10-year PREVENT risk) is the locus of greatest clinical ambiguity and the focus of my principal recommendation. Rather than treating this range as a single shared-decision zone, I propose explicit, rule-based subdivision by age, sex, and the presence of metabolic syndrome or MASLD. The rationale is that a 10-year estimate compresses very different lifetime trajectories. A younger patient with metabolic syndrome and MASLD may have a modest 10-year but a high 30-year risk, a divergence now directly quantifiable with the PREVENT 30-year estimate and formally recognized by the 2026 guideline as a treatment consideration in its own right (30, 33), whereas an older patient may cross the treatment threshold on age alone. Sex modifies both underlying risk and statin-attributable diabetes burden. Metabolic syndrome and MASLD identify the subset with simultaneously higher cardiovascular risk and greater statin-associated diabetes risk, demanding individualized rather than algorithmic judgment (14, 20, 23).

**Figure 2:**
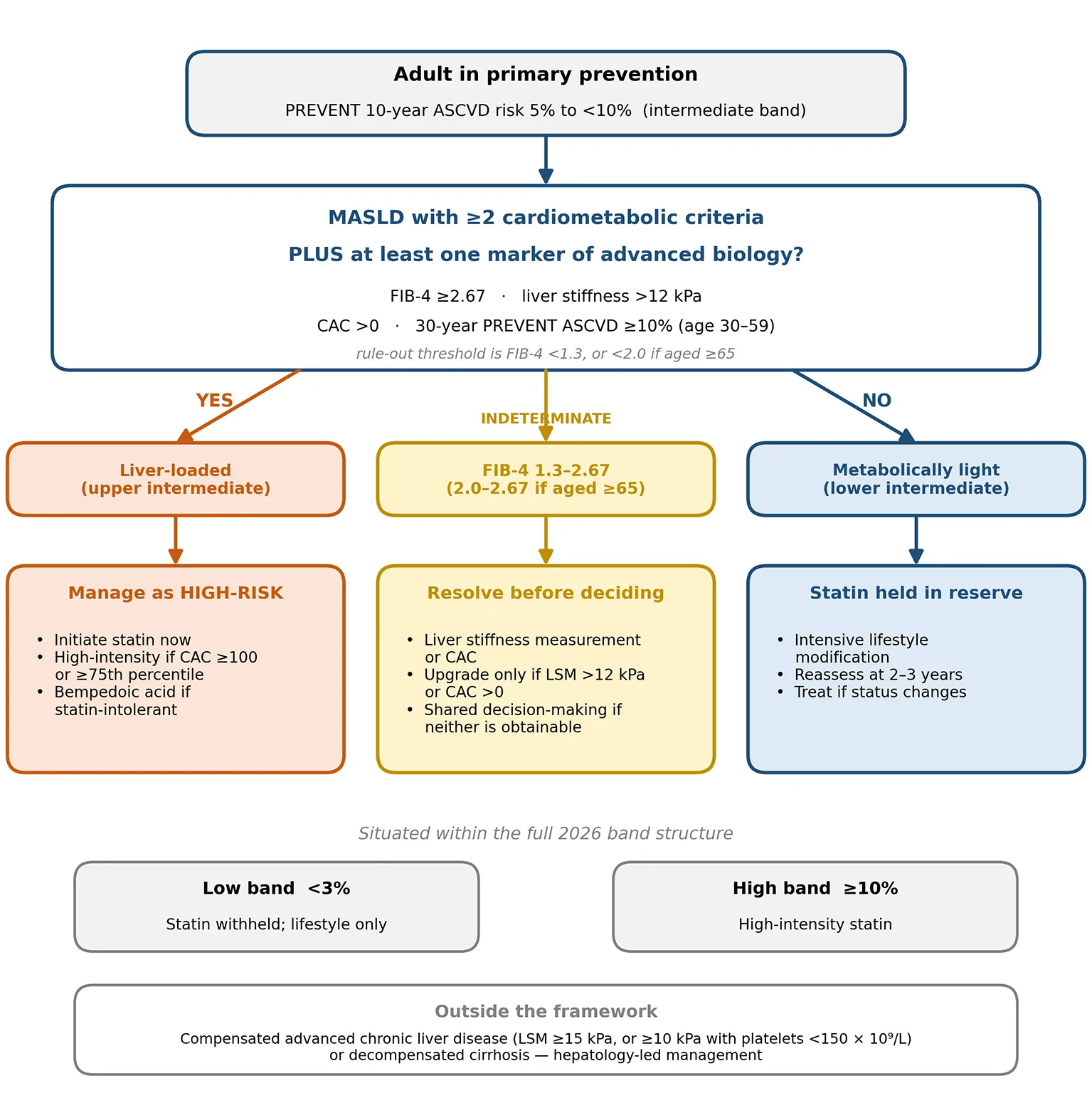
Fibrosis-informed upgrade rule for the 2026 PREVENT intermediate stratum. An adult in the intermediate band (PREVENT 10-year ASCVD risk 5% to < 10%) is reclassified by the presence of MASLD meeting two or more cardiometabolic criteria together with at least one marker of advanced biology, defined as a FIB-4 of 2.67 or above, a liver stiffness measurement above 12 kPa, a coronary artery calcium score above zero, or a 30-year PREVENT ASCVD risk of 10% or more at ages 30–59. The rule-out threshold is a FIB-4 below 1.3, or below 2.0 in adults aged 65 years or older; an indeterminate FIB-4 between these values and 2.67 is resolved by liver stiffness measurement or coronary artery calcium rather than acted upon directly. Liver-loaded patients in the upper intermediate band are managed as high-risk, whereas metabolically light, low-fibrosis patients in the lower intermediate band default to lifestyle therapy with pharmacotherapy held in reserve. The lower panel situates the rule within the full 2026 band structure and states the disease severity beyond which the framework does not apply. CAC, coronary artery calcium; FIB-4, fibrosis-4 index; LSM, liver stiffness measurement; MASLD, metabolic dysfunction-associated steatotic liver disease; PREVENT, Predicting Risk of cardiovascular disease EVENTs. The rule's exposure is a noninvasive fibrosis risk category in a patient meeting the MASLD definition; steatohepatitis is not a variable in the rule.

Operationally, the hypothesis takes the form of the following upgrade rule, every term of which is defined quantitatively in Table 3. A patient in the intermediate band who has MASLD meeting two or more of the five cardiometabolic criteria of the 2023 multisociety nomenclature (6), together with at least one marker of advanced biology, should be managed as high-risk, with statin initiation rather than indefinite deferral, and with high-intensity therapy when CAC is ≥100 or ≥75th percentile. A marker of advanced biology is defined as any one of: FIB-4 ≥2.67 (age-adjusted as specified); liver stiffness measurement >12 kPa; CAC >0; or a 30-year PREVENT ASCVD risk ≥10% in an adult aged 30–59. An indeterminate FIB-4 (1.3–2.67, or 2.0–2.67 at age ≥65) does not by itself satisfy this condition and must be resolved by liver stiffness or CAC. The lower, metabolically light band of the same stratum, defined by intermediate 10-year risk with FIB-4 below the age-appropriate rule-out threshold, CAC of zero where measured, and fewer than two cardiometabolic criteria, may reasonably default to intensive lifestyle modification with reassessment at 2–3 years, and pharmacotherapy held in reserve. In this way, ambiguity in the middle is resolved not by an unstructured conversation but by a small number of clinically meaningful, often liver-derived, modifiers, as summarized in Figure 2.

### 7.3 A worked example in which fibrosis changes management independently of coronary calcium

Consider two adults, both aged 52, both with a PREVENT 10-year risk of 8%, and both in a setting where coronary computed tomography is not reimbursed for primary prevention and has been declined; this is the ordinary situation across most of Asia and much of Europe, and it is the situation in which a zero-cost marker has to carry the decision. The first patient has a body-mass index of 24 kg/m^2^, normal blood pressure, normal triglycerides, no dysglycaemia, and a FIB-4 of 0.9. She satisfies neither the metabolic nor the fibrosis limb of the rule; published modeling places the benefit-to-harm balance for such a patient toward the lower end of the LHH range of roughly 2.3 to 2.9 (22), and the appropriate action is intensive lifestyle modification with reassessment in 2–3 years. The second patient has a waist circumference of 98 cm, a blood pressure of 138/86 mmHg, triglycerides of 2.1 mmol/L, hepatic steatosis on ultrasound, and a FIB-4 of 2.9. He meets three cardiometabolic criteria and the FIB-4 rule-in threshold, and is therefore managed as high-risk with statin initiation now rather than at a reassessment years hence. Neither decision used a calcium score, and neither could have been reached from the PREVENT output alone, which was identical in the two patients.

A third patient makes the discordant case concrete. A 58-year-old with the same 8% 10-year risk, two cardiometabolic criteria, a FIB-4 of 2.8, and a calcium score of zero should not, on the framework proposed here, be started on a statin: the zero score governs, and deferral is correct (33). The elevated FIB-4 nonetheless changes three things, namely referral to the hepatology fibrosis pathway, a reassessment interval of 2–3 years instead of five, and explicit attention to incident heart failure, which the calcium score does not address and which is part of the PREVENT composite (40). Finally, for the rare upgraded patient who cannot tolerate a statin, a diabetes-neutral agent such as bempedoic acid preserves the benefit while avoiding the harm that the metabolic phenotype would otherwise amplify (44). These illustrations are drawn from the cited modeling and guideline sources rather than from a dedicated trial, and they define the quantity that future work should measure directly.

### 7.4 Define and protect the low-risk stratum

The low-risk stratum should be explicitly defined and actively defended. A patient with a 10-year PREVENT risk below 3%, a CAC score of zero where measured, a FIB-4 below the age-appropriate rule-out threshold, no risk-enhancing factors, and no metabolically loaded MASLD should not, as a rule, receive pharmacotherapy; lifestyle modification is the intervention, and statins are withheld unless reassessment moves the patient into a higher band. This is the stratum that undifferentiated prescription would harm most, converting low-benefit individuals into recipients of cost, polypharmacy, and a small but real diabetes risk in exchange for negligible absolute gain. Defining low risk clearly is not therapeutic nihilism; it is the necessary counterpart to boldness at the high end of the distribution. The refined scheme is summarized in Table 4.

**Table 4:** A liver-aware refinement of cardiovascular risk strata for statin allocation (2026 PREVENT-based bands).

Stratum	PREVENT 10-yr risk	Liver/metabolic modifiers	Recommended action
Low	< 3%	FIB-4 below age-appropriate rule-out threshold; < 2 cardiometabolic criteria; CAC 0 where measured	Lifestyle only; statin withheld; reassess at 5 years or on change of status
Borderline	3% to < 5%	Standard risk-enhancing factors govern; MASLD with ≥2 cardiometabolic criteria plus a marker of advanced biology counts as a risk enhancer	Lifestyle; statin considered if risk enhancers present, or if CAC >0
Intermediate – lower	5% to < 10%	FIB-4 below age-appropriate rule-out threshold; CAC 0 where measured; < 2 cardiometabolic criteria	Intensive lifestyle; reassess at 2–3 years; CAC or fibrosis-guided; pharmacotherapy held in reserve
Intermediate – indeterminate	5% to < 10%	MASLD with ≥2 cardiometabolic criteria but FIB-4 in the indeterminate zone (1.3–2.67; 2.0–2.67 if age ≥65) and no other marker	Resolve with LSM or CAC before deciding; shared decision-making if neither is obtainable
Intermediate – upper (liver-loaded)	5% to < 10%	MASLD with ≥2 cardiometabolic criteria PLUS ≥1 marker of advanced biology (FIB-4 ≥2.67, LSM >12 kPa, CAC >0, or 30-yr PREVENT ASCVD ≥10% at ages 30–59)	Manage as high-risk: initiate statin; high-intensity if CAC ≥100 or ≥75th percentile; bempedoic acid if statin-intolerant
High	≥10%	MASLD with advanced fibrosis adds weight but does not change the indication	High-intensity statin
Very-high	Clinically defined	Recurrent/multivessel ASCVD, FH, DM with target-organ damage, very-high-risk CKD; CKM stage 3–4; advanced fibrosis + DM	Lowest LDL-C targets; early ezetimibe/PCSK9i combination
Outside the framework	Any	Compensated advanced chronic liver disease (LSM ≥15 kPa, or ≥10 kPa with platelets < 150 × 10?/L) or decompensated cirrhosis (Child–Pugh B or C, ascites, variceal bleeding, encephalopathy)	Hepatology-led management; statins remain safe in compensated disease but the competing-risk calculus in section 7.5 applies

ASCVD, atherosclerotic cardiovascular disease; CAC, coronary artery calcium; CKD, chronic kidney disease; CKM, cardiovascular-kidney-metabolic; DM, diabetes mellitus; FH, familial hypercholesterolemia; FIB-4, fibrosis-4 index; LDL-C, low-density lipoprotein cholesterol; LSM, liver stiffness measurement; MASLD, metabolic dysfunction-associated steatotic liver disease; PCSK9i, PCSK9 inhibitor; PREVENT, Predicting Risk of cardiovascular disease EVENTs. The table is a conceptual proposal intended to prompt individualized judgment and prospective validation, not a validated decision rule.

### 7.5 Boundaries: where the rule does not apply, and why competing risk sets them

The logic that makes fibrosis useful in early disease inverts at the advanced end of the fibrosis distribution, and the boundary must be stated in operational terms rather than gestured at. The reason is competing risk. In a meta-analysis of biopsy-proven NAFLD cohorts, liver-related mortality rose steeply with fibrosis stage, with mortality rate ratios relative to stage 0 of 16.69 (95% CI 2.92–95.36) at stage 3 and 42.30 (95% CI 3.51–510.34) at stage 4, while all-cause mortality rate ratios were 3.48 (2.51–4.83) and 6.40 (4.11–9.95) respectively; the confidence intervals around the liver-related estimates are very wide, reflecting a pooled sample of only 1,495 patients, so the gradient should be read as directionally robust but quantitatively imprecise (47). That analysis also states that it could not adjust for comorbid conditions or demographic factors, and that its stage 0 reference group included patients with simple steatosis and with steatohepatitis without fibrosis, so the comparison is a fibrosis gradient rather than a phenotype-pure one (47). Two consequences follow. First, in advanced fibrosis a growing fraction of deaths is liver-related and therefore not preventable by lipid lowering, so the absolute cardiovascular benefit of a statin over a 10-year horizon is smaller than a cardiovascular risk equation, which does not model liver-related death as a competing event, will estimate. Second, the same patients have the shortest expected survival, which further compresses the time available for a preventive therapy whose benefit accrues over years. The net effect is that the fibrosis-as-risk-enhancer logic, which correctly increases the intensity of cardiovascular management across the early and intermediate fibrosis range, ceases to hold once fibrosis is advanced enough for liver-related death to dominate.

I therefore specify the boundary as follows. The upgrade rule applies to adults in the 5% to < 10% band with steatotic liver disease up to and including indeterminate or moderate fibrosis. It does not apply, and cardiovascular management should be led jointly with hepatology rather than by the rule, once a patient meets criteria for compensated advanced chronic liver disease, operationally an LSM of 15 kPa or more, or 10 kPa or more with a platelet count below 150 × 10?/L, or established cirrhosis on imaging or biopsy. It is explicitly suspended in decompensated cirrhosis, operationally Child–Pugh class B or C, or any history of ascites, variceal hemorrhage, or hepatic encephalopathy, where statin pharmacokinetics, myopathy risk, and competing mortality are all altered and where society guidance already counsels caution (8, 15). Between these poles, in compensated advanced disease without decompensation, statins remain safe and a cardiovascular indication should still be honored; what changes is that the fibrosis marker no longer functions as a reason to escalate, because it has become a reason to model competing risk explicitly.

## 8 A validation agenda: from structured reasoning to testable rule

Because the central proposal is a refinement of clinical judgment rather than a rule derived from a single trial, it should be stated in a form that can be tested and, in principle, refuted. The hypothesis is precise: among adults in the 5% to < 10% PREVENT band, the addition of liver-derived and imaging modifiers, comprising cardiometabolic-criterion count, FIB-4 or liver stiffness, CAC, and 30-year PREVENT risk, identifies a subgroup whose observed event rate approaches that of the conventional high-risk stratum and in whom statin initiation yields net benefit.

Endpoint choice requires care, because the PREVENT equations estimate both a total cardiovascular disease outcome that includes heart failure and a narrower atherosclerotic outcome, and the 2026 allocation bands are drawn on the atherosclerotic estimate. Validation should therefore prespecify both. Total cardiovascular disease should serve as the primary endpoint, because it captures the heart-failure hazard with which noninvasive fibrosis scores are most consistently associated in prospective data (40) and because it is the broader outcome the equations were built around; atherosclerotic cardiovascular disease should serve as a co-primary endpoint, because that is the scale on which the treatment bands, and therefore the clinical decision, are defined. Reporting only one of the two would leave either the reclassification statistics or the treatment implication uninterpretable, which is the misalignment the present proposal must avoid.

Four predictions follow, and each is falsifiable. First, within the intermediate band, event rates should rise monotonically across fibrosis categories, such that liver-loaded patients approach high-risk event rates. Second, and this is the decisive test of incremental value, adding FIB-4 or LSM to PREVENT should improve discrimination and calibration and yield a positive continuous net reclassification improvement within the band; if the change in the C-statistic is negligible and the net reclassification improvement does not differ from zero after adjustment for the PREVENT covariates, the hypothesis is refuted. Third, in the subgroup with CAC available, the fibrosis markers should retain independent association after adjustment for the calcium score, and the reclassification analysis should be repeated with CAC in the base model; if fibrosis adds nothing once CAC is known, the framework should be restricted explicitly to settings where CAC is unavailable. Fourth, the LHH should be more favorable in the upgraded subgroup than in the metabolically light subgroup at the same nominal 10-year risk. A further condition applies across all four: if decision-curve analysis shows no net benefit relative to treating all or treating none within the band across threshold probabilities of 3% to 10%, the rule should be abandoned irrespective of any statistically significant association (48).

These predictions are testable in existing prospective MASLD and population cohorts that capture the relevant variables, provided the design is specified in advance. Participants should be adults aged 30 to 79 in the 5% to < 10% band with no prevalent cardiovascular disease at baseline, which is the population in which the equations were derived (30). The index date should be the date of the complete biochemical panel from which FIB-4 is computed, with a blanking period before follow-up begins and a landmark analysis reported, so that events present at baseline are not counted as incident. The method by which steatosis was ascertained should be recorded and reported, and cohorts should not be pooled across methods without a sensitivity analysis, since ultrasonography, computed tomography, magnetic resonance proton density fat fraction, controlled attenuation parameter, and validated indices differ in sensitivity at low fat fractions. A subset with elastography or histology should be included so that the noninvasive fibrosis classification can be calibrated against a reference standard within the same population, and the distribution of fibrosis category should be reported for the intermediate band specifically. Steatohepatitis should not be used as a classifier, and alcohol intake should be quantified so that MASLD and MetALD can be separated.

Endpoints should be defined and ascertained to a cardiological standard. Each component of the composite should be defined *a priori* with its diagnostic code list published. In prospective cohorts, events should be adjudicated by a committee applying standardized definitions and blinded to the fibrosis marker; where only administrative data exist, validated case-identification algorithms should be used with a chart-reviewed sample and a reported positive predictive value. Outcome ascertainment should in all cases be blinded to FIB-4 and liver stiffness.

The minimum adjustment set is age, sex, diabetes and glycated hemoglobin, systolic blood pressure and antihypertensive use, LDL cholesterol, HDL cholesterol, triglycerides and lipid-lowering therapy, body-mass index and waist circumference, smoking, and estimated glomerular filtration rate with urinary albumin-to-creatinine ratio, with prevalent cardiovascular disease handled as an exclusion rather than a covariate. Three further variables are required by this particular exposure: quantified alcohol intake, which separates MASLD from MetALD and independently affects both outcomes; statin and lipid-lowering exposure modeled as time-varying, since the analysis concerns an untreated risk marker in a progressively treated population; and a socioeconomic deprivation measure. The primary specification should adjust for the PREVENT linear predictor entered as an offset rather than for its components one at a time, because the question is incremental value over the equation as it is actually used; a component-wise model should be retained as a sensitivity analysis, and a quantitative bias analysis for unmeasured confounding should be reported. No adjustment converts an observational association into evidence of independence in the causal sense, and the results should be presented on that understanding.

The analysis must be a competing-risks analysis in the formal sense. Cumulative incidence should be estimated with a subdistribution model in which liver-related death and non-cardiovascular death are competing events and incident T2D is carried as a countervailing event, with cause-specific hazard models reported alongside, since the two answer different questions, and with cumulative incidence plotted by fibrosis category within the band, so that the LHH is estimated empirically rather than modeled and the benefit attenuation predicted in section 7.5 is measured rather than assumed. The requirement is not merely one of good practice: the PREVENT equations were themselves developed with adjustment for the competing risk of non-cardiovascular death (30), so a fibrosis-augmented model that ignored competing risk would not be comparable to the equation it is intended to extend.

Incremental value should be reported as the change in the C-statistic with a confidence interval; the calibration slope with a calibration plot by decile of predicted risk and calibration-in-the-large, computed within the 5% to < 10% band rather than across the whole cohort, since a rule that operates in one band may be well calibrated overall and miscalibrated where it acts; the continuous and categorical net reclassification improvement, the latter evaluated at the 5% and 10% boundaries because those are the boundaries that carry the treatment decision (49); the integrated discrimination improvement; and decision-curve analysis reporting net benefit across threshold probabilities of 3% to 10%, which spans the 2026 bands (48). Reporting should follow the TRIPOD statement for prediction-model studies (50). Studies should be sized for the precision of the reclassification and net-benefit estimates rather than for the power to detect a hazard ratio.

Analyses should be sex-stratified, given the differential diabetogenic burden, and stratified by age band, given the age dependence of FIB-4, and should include external validation in East Asian and lean-MASLD cohorts, where the calibration of Western risk equations is least certain. A threshold-optimization analysis should also be prespecified, since the FIB-4 and LSM values proposed here are defaults imported from hepatic diagnosis rather than values derived against a cardiovascular endpoint; the analysis should report the cut-points that optimize the benefit-to-harm trade-off and compare them with the defaults. Until such data exist, the proposal should be applied as structured clinical reasoning, not as a validated algorithm, and Tables 3, 4 should be read in that spirit.

## 9 Discussion

The statin is a risk-stratified therapy, not a universal preventive agent. The same evidence that proves statins work also proves that their value is conditional on absolute risk: relative benefit is nearly constant across the risk spectrum, so absolute benefit, and therefore the justification for lifelong therapy, rises and falls with baseline risk, while harms and costs do not. The 2026 guidelines reinforce this logic by adopting PREVENT, lowering the numeric thresholds, and embedding metabolic and renal biology into risk, and in doing so they make the refinement of the compressed middle stratum more, not less, urgent.

The liver sharpens both halves of the argument. It identifies a large, high-risk, and badly under-treated population in whom clinicians should be considerably bolder, and through MASLD, metabolic burden, fibrosis stage, age, and sex it supplies measurable variables that may subdivide the ambiguous middle. Disease-modifying MASH therapies and the diabetes-neutral agent bempedoic acid enrich, rather than replace, this stratified approach. The contribution of this article is not a further catalog of statin evidence in MASLD, since that literature is mature, but the procedural step it has lacked, namely a fully specified, liver-derived decision architecture for the specific stratum in which the 2026 guidelines leave clinicians most uncertain, expressed as a falsifiable hypothesis with a defined validation design and an explicit boundary of application.

Several limitations follow from the nature of the argument, and three deserve emphasis. First, the proposed thresholds are defaults imported from hepatic diagnosis, not cut-points derived against a cardiovascular endpoint; FIB-4 ≥2.67 is adopted because of its high positive predictive value for prevalent coronary calcification in MASLD, not because any study has shown that it identifies a subgroup with a distinct treatment effect. Second, and most importantly, the incremental value of fibrosis markers over the PREVENT equations, and over coronary artery calcium where the latter is available, is not established; section 8 specifies the analysis that would establish or refute it, and until that analysis exists the framework should be regarded as a hypothesis about how to organize existing information rather than as evidence that new information has been added. Third, the hepatoprotective signal attributed to statins is observational and, as Mendelian randomization suggests, may be partly confounded; the proposal therefore rests on the cardiovascular case for statins, to which the hepatic data add context rather than an independent indication. The calibration of PREVENT outside U.S. cohorts is also unconfirmed, which is both a limitation and, in part, the motivation for anchoring the rule to biologically defined liver markers. These limitations define the research agenda rather than undermine the hypothesis. Finer stratification, not broader dispensation, is the way to capture the benefit that maximalists rightly seek while sparing low-risk individuals the harms they tend to discount; the liver, and the fibrosis marker in particular, is a plausible place for that finer stratification to begin.

Three further limitations follow from the nature of the evidence rather than from the argument. The cut-points are anchored to coronary artery calcification, which is an imaging surrogate, and in the cohort that supplies them the design is cross-sectional and the calcification prevalent, so their performance against adjudicated clinical endpoints is unknown (37). None of the studies supporting them staged fibrosis histologically, so the framework rests throughout on noninvasive risk categories rather than on stages, and Supplementary Table S1 sets out that provenance study by study. And every association on which the framework relies is observational, with the residual confounding that implies. It is worth stating precisely what this does and does not undermine. The hypothesis concerns prediction and allocation, not mechanism, so nothing in it requires fibrosis to be causal: a variable that reclassifies risk needs only to be associated with the outcome and to carry information the existing model lacks. The framework accordingly does not propose fibrosis as a treatment target and makes no claim that reducing fibrosis reduces cardiovascular events.

## Data Availability

The original contributions presented in the study are included in the article/supplementary material, further inquiries can be directed to the corresponding author.
